# Rapid research response to the 2009 A(H1N1)pdm09 influenza pandemic (Revised)

**DOI:** 10.1186/1756-0500-6-177

**Published:** 2013-05-03

**Authors:** Wendy A Keitel, Pedro A Piedra, Robert L Atmar, Gail Demmler, Hana M El Sahly, Jill Barrett, Rebecca A Halpin, Rosanna Lagos, Susan Fisher-Hoch, Flor Munoz

**Affiliations:** 1 Department of Molecular Virology & Microbiology, Houston, TX, USA; 2 Department Medicine, Houston, TX, USA; 3 Department Pediatrics, Baylor College of Medicine, Houston, TX, USA; 4 The EMMES Corporation, Rockville, MD, USA; 5 J. Craig Venter Institute, Rockville, MD, USA; 6 Centro para Vacunas en Desarrollo-Chile, Santiago, Chile; 7 University of Texas School of Public Health, Brownsville, TX, USA; 8 Departments of Molecular Virology & Microbiology and Medicine, Baylor College of Medicine, Room 280, One Baylor Plaza, Houston, TX 77030, USA

**Keywords:** 2009 H1N1 virus, Immune responses, Influenza

## Abstract

**Background:**

When novel influenza viruses cause human infections, it is critical to characterize the illnesses, viruses, and immune responses to infection in order to develop diagnostics, treatments, and vaccines. The objective of the study was to collect samples from patients with suspected or confirmed A(H1N1)pdm09 infections that could be made available to the scientific community. Respiratory secretions, sera and peripheral blood mononuclear cells (PBMCs) were collected sequentially (when possible) from patients presenting with suspected or previously confirmed A(H1N1)pdm09 infections. Clinical manifestations and illness outcomes were assessed. Respiratory secretions were tested for the presence of A(H1N1)pdm09 virus by means of isolation in tissue culture and real time RT-PCR. Sera were tested for the presence and level of HAI and neutralizing antibodies against the A(H1N1)pdm09 virus.

**Findings and conclusions:**

Thirty patients with confirmed A(H1N1)pdm09 infection were enrolled at Baylor College of Medicine (BCM). Clinical manifestations of illness were consistent with typical influenza. Twenty-eight of 30 had virological confirmation of illness; all recovered fully. Most patients had serum antibody responses or high levels of antibody in convalescent samples. Virus-positive samples were sent to J. Craig Venter Institute for sequencing and sequences were deposited in GenBank. Large volumes of sera collected from 2 convalescent adults were used to standardize antibody assays; aliquots of these sera are available from the repository. Aliquots of serum, PBMCs and stool collected from BCM subjects and subjects enrolled at other study sites are available for use by the scientific community, upon request.

## Background

Influenza is a highly contagious acute respiratory infection that affects all age groups but has significant morbidity and mortality, especially in the very young and elderly populations. Influenza is usually a seasonal disease with high attack rates, short incubation period and rapid transmission. Periodic infections of humans with novel strains of influenza raise concerns that a pandemic of influenza could be unfolding. Novel influenza A viruses, including H5N1, H7N7, and H9N2 viruses, have produced human infections in recent years without evolving into a pandemic. However, in the spring of 2009 a novel influenza A/H1N1 virus [A(H1N1)pdm09] emerged in Mexico, followed by recognition of international spread to the US [1,2], A pandemic caused by the A(H1N1)pdm09 virus was declared by the World Health Organization on June 11, 2009 [3]. When novel viruses cause infections in humans, it is critical to obtain samples as soon as possible to identify and characterize the viruses and the disease they cause, and to understand the host immune response to infection. These samples are necessary to develop diagnostic tests, treatments, and vaccines for prevention of infection.

The purpose of this study was to collect blood and respiratory samples from subjects who were known or suspected to have infection caused by the A(H1N1)pdm09 virus, and to make available to the scientific community new strains of influenza viruses and other immunologic reagents to facilitate influenza research. These samples were used to detect and isolate viruses for further characterization and to study the adaptive immune responses following infection. The purpose of this manuscript is to describe the clinical and laboratory features of infection among 30 subjects enrolled at Baylor College of Medicine (BCM) who had confirmed A(H1N1)pdm09 infection, and to inform the research community about the availability of samples collected from subjects enrolled at several study sites, as well as other A(H1N1)pdm09 resources available through the National Institutes of Health (NIH).

## Methods

### Subjects

Male and female subjects of all ages (> 1 day old) who had an influenza-like illness (subjects who had at least one respiratory symptom and at least one of the following: oral temperature ≥ 100°F; feeling feverish; and/or close contact with a confirmed case), or who had current or recent laboratory-confirmed A(H1N1)pdm09 influenza virus infection were invited to participate in the study. Exclusion criteria included inability or unwillingness of the subject (or parent/guardian) to provide informed consent; influenza known to be caused by a strain other than the A(H1N1)pdm09 virus; and participation in another blood donation study. Subjects were recruited from local healthcare facilities or referred by their health care providers or through word of mouth. The study was conducted in accordance with protocols approved by the BCM Institutional Review Board.

### Clinical procedures

Adults provided written informed consent; written parental informed consent was obtained for subjects under the age of 18 years. After obtaining informed consent, information regarding the patient’s potential source of infection (exposures) and the illness was collected, along with pertinent medical history and demographic information. Permission was requested to review the subjects’ medical records for additional clinical and laboratory information (e.g., treatment regimen, results of rapid antigen detection tests and other laboratory results, if applicable). A targeted physical examination was performed based on the subject’s history and/or signs and symptoms at the time of enrollment. Samples were collected for virus isolation and for evaluation of immune responses. Subjects (or parents/guardians) were given the option to refuse donation of some of the samples, if desired. Combined nasal wash/throat swab (NW/TS) specimens were collected for detection and isolation of A(H1N1)pdm09 influenza viruses.

Venous blood samples for sera and peripheral blood mononuclear cells (PBMCs) were collected to assess immune responses following infection. The amount of blood collected varied according to the age of the subject and whether the illness was acute or convalescent: 5 – 10 mL from infants; 20 – 60 mL from children; and up to 120 mL was collected from adults. Subjects (and parents/legal guardians) were asked for their permission to be contacted for up to twelve weeks after initial sampling for collection of additional information and samples.

The types and frequencies of subsequent sample collection were based on the time since illness onset and subject consent. NW/TS samples for virus identification were collected up to 2 weeks after illness onset. If any sample was positive by PCR, then respiratory samples were collected again approximately 5 days after the most recent PCR-positive sample until the results were negative. For example, if the initial sample collected 2 days after illness onset was positive, then the subject was re-sampled at 4–9 days, 10–14 days, and 15–19 days after illness onset or until the PCR became negative. Acute blood samples for serum and PBMCs were collected within 2 weeks of illness onset, and convalescent blood samples were collected up to 35 days after illness onset.

Symptoms were graded on a scale of 0 to 3, where 0 was absent; 1 was mild (did not interfere with activity); 2 was moderate (interfered somewhat with activity); and 3 was severe (incapacitating). Fever was graded on a scale of 0–3, where 0 was absent; 1 was mild (100°F-101.1°F); 2 was moderate (101.2-102°F); and 3 was severe (≥ 102.1°F). Vomiting was graded as follows: 0 = No vomiting; 1 = 1–2 episodes/24 hrs; 2 = ≥ 2 episodes/24 hrs; and 3 = Requires IV hydration. The severity of diarrhea was graded as follows: 0 = 0–1 loose stools/24 hrs; 1 = 2–3 loose stools/24 hrs; 2 = 4–5 loose stools/24 hrs; and 3 = ≥ 6 loose stools/24 hours or requires IV hydration.

### Laboratory procedures

#### Virus detection and isolation

NW/TS samples collected from subjects presenting within 2 weeks of onset of illness were tested for A(H1N1)pdm09 influenza virus using a real time, reverse transcriptase polymerase chain reaction (rtRT-PCR) platform with CDC published primers [4]. In brief, RNA was extracted from NW/TS specimens with the QIAamp Viral RNA Mini Kit (Qiagen) in the semiautomated QIAcube unit. Specimens were tested by rtRT-PCR using specific primers and fluorescent Taqman probes for the novel 2009 influenza A/H1N1 and for seasonal influenza A and B viruses. In addition, NW/TS samples were inoculated into primary rhesus monkey kidney (RhMK) tubes for virus isolation. Hemadsorption (HA) with guinea pig red blood cells (0.25%) was used to identify the presence of hemadsorbing viruses including the A(H1N1)pdm09 influenza virus. HA-positive harvests were tested by rtRT-PCR to identify the A(H1N1)pdm09 influenza virus.

#### Serum HAI and neutralizing antibody assays

Assays for A(H1N1)pdm09 -specific serum hemagglutination-inhibiting (HAI) and neutralizing antibodies were performed as described previously [5]. A fourfold or greater rise in HAI and/or neutralizing antibody titers between the acute and convalescent serum samples was considered significant and consistent with a recent infection. Infection was considered confirmed if at least one of the following criteria was met: positive rtRT-PCR or culture on respiratory samples and/or ≥4-fold increase in HAI or neutralizing antibody titer.

## Findings

**Figure 1:**
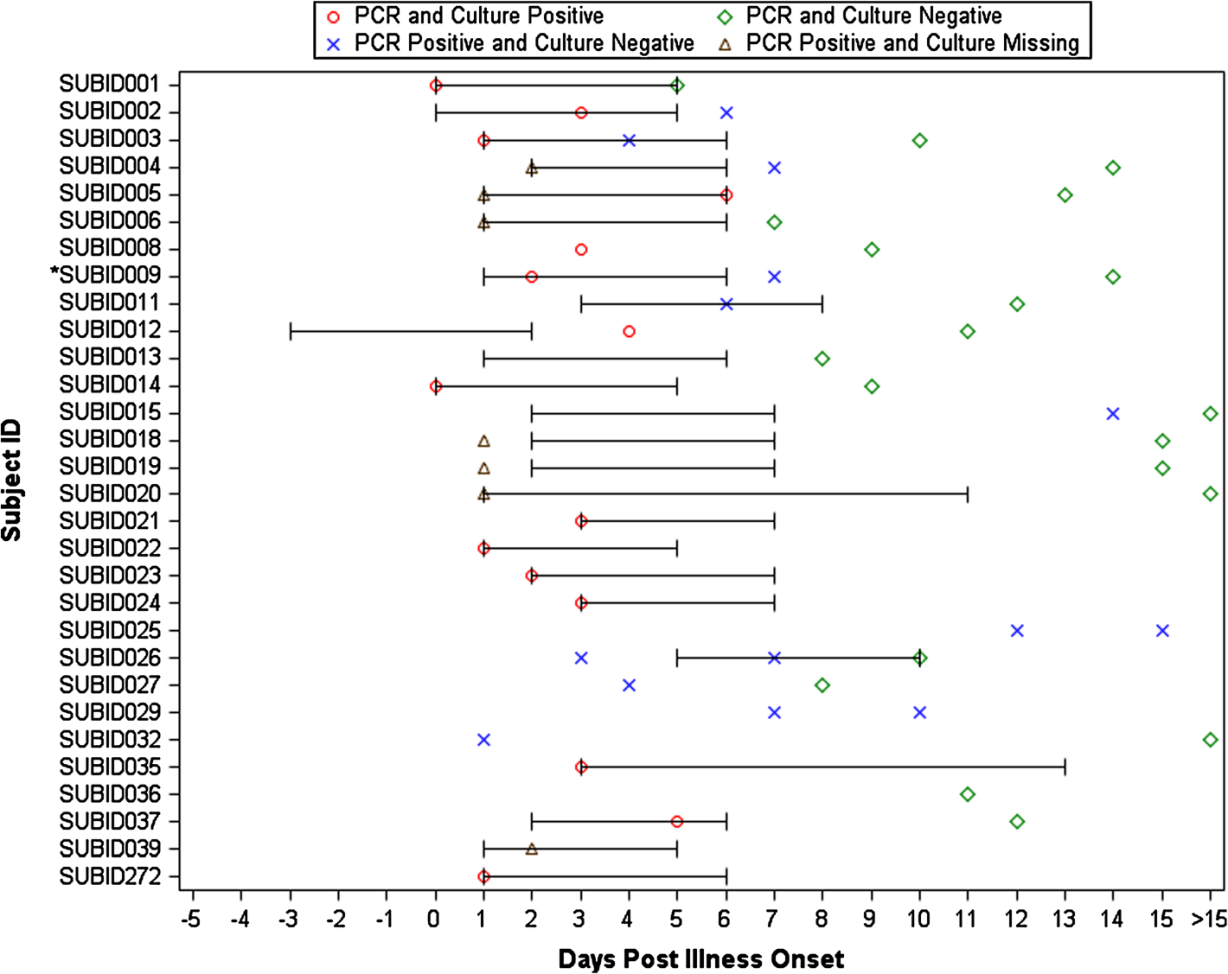
**Virus shedding among patients with confirmed A(H1N1)pdm09 infections.** Note: The bracketed line indicates the period during which oseltamivir was taken. Asterisk (*) indicates the subject (SUBID009) had a negative culture result at Day 1; PCR result not available for Day 1.

Forty-one subjects were enrolled at BCM between May 2009 and July 2009. One enrolled child declined to provide samples and is not included in the analyses. Seven other subjects had no virological confirmation of infection: three children with a negative rtRT-PCR test on respiratory samples had no blood samples collected; and four adults had no detectable HAI or neutralizing antibody in acute (N=4) or convalescent (N=3) samples. The NW/TS samples on these 4 adults were negative for A(H1N1)pdm09 influenza virus. Of the remaining 33 subjects, 28 had virological confirmation of A(H1N1)pdm09 infection (Figure 1). Eighteen of these were confirmed in local hospital or health department laboratories prior to enrollment in the study, and 13 had rtRT-PCR positive samples after enrollment. Two additional subjects (SUBID013 and SUBID036) had negative rtRT-PCR and culture 9 and 12 days after illness onset, respectively (both with confirmed infections in the household); both of these subjects had significant increases in serum HAI and/or neutralizing antibody titers. The age distribution of the 30 subjects who had confirmed infections was as follows: 5 were < 5 years old; 16 were 5–9 years old; 4 were 10–17 years old; and 5 were > 18 years old.

Three other subjects without virological confirmation had serological evidence of probable infection: 2 subjects who had high titers of antibody (HAI and/or neutralizing antibody titer ≥ 1280) on a single specimen collected 11 and 16 days after illness onset and had household contact with a confirmed case (the mother and grandmother of subject SUBID032); and one with HAI and neutralizing antibody titers of 28 and 57, respectively, on day 12 of illness. These 3 subjects are not included in the analyses.

Demographic features of the 30 subjects with confirmed infections are shown in Table 1. An equal number of males and females with confirmed infections were enrolled; most were white. The mean age of these subjects was 12.1 years (range=2 months to 53 years). Seven subjects (23%) reported a history of asthma, and one subject had a history of hypertension. No subjects reported a history of diabetes mellitus, current pregnancy, HIV infection, cancer, immunosuppression, tobacco use or other significant neurological, kidney, liver, or hematological disorders. Twenty subjects had received a seasonal influenza vaccine during the fall of 2008.

**Table 1:** Demographic characteristics of subjects with confirmed A(H1N1)pdm09 infections

		
**Sex**	Male	Female
	15 (50) *	15 (50)
**Ethnicity**	Non-Hispanic	Hispanic
	22 (73)	8 (27)
**Race**	White	Asian, Black, or Multiracial
	27 (90)	1 (3) each
**Age (years)**	Mean (SD)	Median (Range)
	12.1 (10.5)	9.1 (2 months, 53 years)

* Number (percent).

### Virological and serological features of infection

The results of sequential culture and PCR assays of respiratory secretions are shown in Figure 1. A bracketed line indicates the period during which antiviral therapy with oseltamivir was administered. Most subjects were treated: 21/30 received a 5-day course of therapy (dosage based on weight) that was started within 3 days after symptom onset, with the exception of one subject whose treatment was started on day 5 of illness. One additional subject developed culture-positive symptomatic disease while receiving antiviral prophylaxis (SUBID012; 75 mg bid × 5 days). Two other subjects received 10 days of antiviral medication (one prophylaxis dosage and one treatment dosage), and 6 were not treated. Among subjects from whom sequential samples were collected, virus was detected by rtRT-PCR in NW/TS samples for up to 15 days after symptom onset. Fourteen subjects (47%) were culture-positive when initially tested, and approximately one-third of rtRT-PCR-positive samples collected after enrollment yielded virus in tissue culture. One subject with a negative culture on day 2 of illness became culture-positive on day 3 (SUBID009). All culture-positive samples were collected within the first week of illness, and all culture-positive samples were also positive by rtRT-PCR. None of the isolates for which sequence data are available had mutations indicating resistance to oseltamivir or zanamivir, including isolates recovered while on therapy (SUBID002, SUBID005, SUBID009 and SUBID037) and the isolate collected from subject SUBID012 after failed prophylaxis. No sequence data are available from the day 14 and 15 samples collected from subjects SUBID015 and SUBID025, respectively.

Serum HAI and neutralizing antibody responses are shown in Table 2. Of the 30 subjects with confirmed infections, 26 provided acute and/or convalescent blood samples for antibody assays: 15 provided acute and convalescent samples; 10 presented at least 2 weeks after illness onset and donated convalescent sample(s) only; and 1 subject provided only an acute sample. Eleven (73%) of those with paired samples developed ≥4-fold rise in serum HAI and/or neutralizing antibody titer; and 3 additional subjects with paired samples had an HAI and/or neutralizing antibody titer of at least 20 in one or both samples. Twenty of 26 subjects (77%) had an HAI and/or neutralizing antibody titer ≥ 40 in acute and/or convalescent sample(s). Only 1 subject failed to develop a convalescent HAI or neutralizing antibody titer ≥ 20 after infection.

**Table 2:** Serum antibody responses among subjects with confirmed A(H1N1)pdm09 infections

**Serologic status**	**Group size**	**HAI antibody**	**Neutralizing antibody**
≥4-Fold Rise in Titer (Acute + convalescent sera available)	15	8	11
Acute Titer ≥ 20; No Significant Rise in Titer	15	6	2
Convalescent Titer ≥ 20 (No acute sample)	10	9	9
Titer < 20 in All Sample(s)	26	2	2
Titer ≥ 40 in Acute and/or Convalescent Sample	26	18	20
No Data	4	4	4

### Clinical features of confirmed infections

Most subjects (N=23) reported close contact with a confirmed or probable case prior to the onset of illness; most of these (N=17) were contact with a classmate in school or daycare. Three were exposed to an infected household member; one each reported exposure to a co-worker, occupational exposure, or contact with multiple cases. Seven subjects could not identify a likely source of their infection. Among the 30 subjects with confirmed infections, two separate clusters were identified. One was an outbreak of A(H1N1)pdm09 influenza in a local elementary school, and the other was an outbreak among students studying and working together at a regional university.

Symptoms and signs reported by subjects with confirmed A(H1N1)pdm09 infections are summarized in Table 3. The most common symptom was cough, reported by 29/30 subjects. The median duration of cough was 10 days; however, cough persisted for up to 32 days after the onset of illness for one subject. Twenty-eight (93%) subjects reported fever. Feverishness, chills, fatigue, lethargy, myalgia, headache, rhinitis, and sore throat were reported by a majority of subjects. Nausea, diarrhea and vomiting were reported by 40%, 23% and 20%, respectively.

**Table 3:** Clinical features of confirmed A(H1N1)pdm09 infections

**Symptom**	**Severity**					**Duration**	
	**None**	**Mild**	**Moderate**	**Severe**	**Any**	**Duration (days) Mean**	**Duration (days) Median (Min, Max)**
Elevated Temperature (Max)	2 (7) *	8 (27)	8 (27)	12 (40)	28	3.2	3.0 (1, 8)
Feverishness	3 (10)	6 (20)	13 (43)	8 (27)	27	3.6	3.0 (1, 6)
Fatigue (Malaise)	4 (13)	4 (13)	12 (40)	10 (33)	26	5.4	4.0 (2, 16)
Irritability	19 (63)	6 (20)	3 (10)	2 (7)	11	4.6	4.0 (2, 15)
Lethargy	15 (50)	3 (10)	7 (23)	5 (17)	15	4.5	4.0 (2, 10)
Myalgia/Body ache	13 (43)	6 (20)	9 (30)	2 (7)	17	3.9	3.0 (2, 7)
Headache	6 (20)	7 (23)	11 (37)	6 (20)	24	3.1	3.0 (1, 6)
Stuffy/Runny nose	11 (37)	11 (37)	8 (27)	0	19	8.4	6.0 (1, 23)
Nausea	18 (60)	7 (23)	5 (17)	0	12	3.0	3.0 (1, 6)
Vomiting	24 (80)	3 (10)	2 (7)	1 (3)	6	1.8	1.5 (1, 4)
Diarrhea	23 (77)	5 (17)	1 (3)	1 (3)	7	3.3	3.0 (1, 10)
Chills	11 (37)	6 (20)	10 (33)	3 (10)	19	2.9	3.0 (1, 7)
Sore throat	14 (47)	9 (30)	6 (20)	1 (3)	16	3.5	3.0 (1, 7)
Cough	1 (3)	11 (37)	16 (53)	2 (7)	29	11.2	10.0 (4, 32)
Shortness of breath	25 (83)	4 (13)	1 (3)	0	5	4.0	4.0 (2, 6)
Wheezing	25 (83)	4 (13)	1 (3)	0	5	3.4	3.0 (1, 6)
Earache	29 (97)	0	1 (3)	0	1	7.0	7.0 (7, 7)
Sinus fullness/Facial pain	27 (90)	2 (7)	1 (3)	0	3	7.0	5.0 (1, 15)
Other symptoms	11 (37)	8 (27)	6 (20)	5 (17)	19	4.4	3.0 (2, 21)

*Number (percent).

All enrolled subjects recovered from their infection. Table 4 summarizes illness outcomes for subjects with confirmed infections. Most subjects (77%) reported visiting a physician for their illness; three subjects were evaluated in the emergency room. One 24-year-old male who had a history of childhood asthma experienced a syncopal episode on day 4 of illness (SUBID025). Evaluation in a local emergency room (ER) was consistent with a vasovagal episode secondary to dehydration. The same subject was evaluated in the ER 4 days later with a complaint of hemoptysis attributed to acute bronchitis. Initial chest radiograph demonstrated mild pulmonary vascular congestion, and a follow-up chest radiograph 4 days later was normal. This subject was treated with 2 courses of antibiotics and did not receive antiviral therapy. A rapid test for influenza was negative on day 8 of illness; rtRT-PCR was positive on respiratory samples on days 12 (time of study enrollment) and 15 after illness onset. One 7-year-old child with asthma was seen in the emergency room for a high fever (104.1°F) and ‘talking nonsense in her sleep’; she was treated with oseltamivir and amoxicillin. Finally, one 2-month-old female infant was evaluated in the ER for fever. No subjects required hospitalization for their illness. Seven subjects did not seek medical attention: 4 of these were part of the 2 clusters of illness; 2 were family members of medically-attended cases; and one was a BCM employee who was aware of the study.

**Table 4:** Clinical outcomes for patients with confirmed A (H1N1)pdm09 infections

**Clinical outcomes**	**N (%)**
Medically Attended Visits	
Physician's office	23 (77)
Emergency room	3 (10)
Missed School or Work	21 (70)
Mean number of days	4
Median (Min, Max)	3 (1, 10)
Medical Diagnoses	
Febrile acute respiratory disease	29 (97)
Otitis media	1 (3)
Bronchitis	1 (3)
Bronchiolitis	1 (3)
Gastroenteritis	3 (10)
Other diagnoses/complications	2 (7)

## Discussion and conclusions

We characterized the clinical, virological and serological responses among 30 ambulatory children and adults, most of whom were previously healthy. The most commonly reported underlying health condition was asthma. Clinical features of illness were similar to those described previously among outpatients who were index cases enrolled in prospective studies of household transmission of A(H1N1)pdm09 infections [6,7]. Of interest was the observation that rtRT-PCR of respiratory secretions remained positive for up to 2 weeks in 2 subjects, one of whom had received a full course of oseltamivir. As noted by others, PCR was considerably more sensitive than culture [8], and remained positive for a longer period of time. While isolates with the H275Y mutation have been detected in patients who developed illness while receiving prophylactic oseltamivir [9], our subjects did not have evidence of this mutation

### Availability of supporting data

The main goal of this study was to collect blood and respiratory samples that could be used to characterize viruses and host immune responses as the A(H1N1)pdm09 pandemic unfolded. Three sites were involved in the sample collection protocol: Baylor College of Medicine; Centro para Vacunas en Desarrollo-Chile; and the University of Texas School of Public Health in Brownsville, TX (see references [10,11] for descriptions of the Brownsville outbreak), During the study a number of viruses were sequenced and the information made publically available in GenBank by late 2009 (GenBank accession numbers for the Houston isolates are CY051903, CY051926, CY052959, CY053173, CY053366, CY087216, CY087223). Selected samples were deposited at the National Institutes of Health (NIH) Biodefense and Emerging Infections Research Resources Repository (BEI). Two adult subjects with moderate to high levels of antibodies in convalescent sera had large volumes of blood collected for the preparation of antibody pools. Aliquots of these pools were used to standardize HAI and neutralizing antibody assays performed on sera collected from participants in clinical trials of A(H1N1)pdm09 vaccines conducted by the NIH and industry. Aliquots of sera used for assay standardization are available upon request from BEI ( http://www.beiresources.org/Catalog.aspx; Catalog Nos. NR-18964 and 189655). Aliquots of PBMCs that can be used for characterization of cellular immune responses to A(H1N1)pdm09 and other influenza viruses have been submitted to the above-mentioned repository. Virus isolates, respiratory samples (nasal wash, throat swab, nasopharyngeal swabs and/or nasal swabs), serum and stool samples collected from A(H1N1)pdm09 -infected subjects and subjects without confirmation of A(H1N1)pdm09 infection are also available for use by qualified investigators, upon request (see Additional file 1, contact dmidresources@niaid.nih.gov for additional information). The numbers and types of samples collected during the study that are available for use by qualified investigators are summarized in Table 5. Finally, additional NIAID-funded A(H1N1)pdm09 reagents/materials are available, as follows: Virus isolates; vaccines (monovalent and trivalent formulations); monoclonal antibodies against HA and NP; polyclonal convalescent human sera; ferret immune sera panels; BPL-inactivated HA; recombinant HA and NA; Gateway clone set (*E. coli*); peptide arrays; and genomic RNAs (HA, NA, NP, matrix, NS1, NS2, PA, PB1 and PB2). These reagents are available by ordering directly from the BEI link shown above. Note that researchers do not need to have NIH funding to access these reagents, and the reagents available in the repository are provided at no cost to the researchers.

**Table 5:** Numbers of subjects from whom the indicated types of samples were collected during the rapid research response study which are available for use by qualified investigators

**H1N1 Infection virologically confirmed?**	**Type of sample**						
	**Acute and convalescent NW, NPS and/or TS, serum and PBMCs**	**Acute and convalescent serum samples, and NW, NPS and/or TS**	**Acute and convalescent NW, NPS and/or TS samples**	**Acute NW, NPS and/or TS; and PBMCs and serum sample**	**Acute NW, NPS and/or TS; +/− acute serum**	**Convalescent serum and PBMCs (> 2 weeks after illness onset)**	**Stool (acute and/or convalescent)**
Yes	43*	19	3	6**	17	41	32
No	8	0	0	34	100	0	2

Abbreviations: *NW*=nasal wash; *NPS*=nasopharyngeal swab; *TS*=throat swab; *PBMC*=peripheral blood mononuclear cells.

*Two of these had significant increases in serum antibody levels without virological confirmation. All subjects have paired sera, but some have only convalescent PBMCs.

**One subject has convalescent PBMCs without serum.

## Competing interests

All authors declare that they have no competing interests.

## Authors’ contributions

WAK: Conception and study design and coordination; data acquisition, data analysis and interpretation; drafting and revising the manuscript. PP: Study design; PCR and culture of viruses; data analysis and interpretation; critical revision of manuscript. RLA: Study design; data analysis and interpretation; critical revision of manuscript. GD: Acquisition of data; PCR and culture of viruses; data analysis and interpretation; critical revision of manuscript. HES: Study design; acquisition of data; critical revision of manuscript. JB: Data analyses; assistance with preparation of tables and figure; critical revision of manuscript. RAH: Viral sequencing; data analysis; critical revision of manuscript. FM: Study design; acquisition of data; critical revision of manuscript. RL: Acquisition of data; critical revision of manuscript. SF-H: Acquisition of data; critical revision of manuscript. All authors have given approval to the final version.

## Supplementary Material

Additional file 1 Specimen listing for rapid research response protocol. Click here for file
